# Improvements in Dental Health and Dentists’ Workload in Norway, 1992 to 2015

**DOI:** 10.1016/j.identj.2021.07.004

**Published:** 2021-09-01

**Authors:** Jostein Grytten, Irene Skau

**Affiliations:** a Department of Community Dentistry, University of Oslo, Oslo, Norway; b Department of Obstetrics and Gynecology, Institute of Clinical Medicine, Akershus University Hospital, Lørenskog, Norway

**Keywords:** Manpower, Dental health, Dental services, Dental policy

## Abstract

**Objectives:**

There has been a marked improvement in dental health in Norway during the last few decades. What effect has this had on provision of dental services, and how has private dental practitioners’ assessment of their workload changed?

**Methods:**

The data were from 2 large surveys of private dental practitioners carried out in 1992 (n = 1056) and 2015 (n = 1237). An analysis of nonresponders showed that they were evenly distributed according to their age, gender, and the region in which their practice was located. Thus, the samples were representative of private dental practitioners. For 1 representative week in practice, the practitioners were asked to report the number of visits and the number of patients who received 1 or more of the following items of treatment: filling, crown, bridge, denture, root filling, extraction, and periodontal treatment. As a measure of patient supply, the responses from the following questions were used: “Based on an overall assessment of economy, workload, and other personal factors, is the number of regular patients adequate? If not, do you wish to have more patients or fewer patients?”

**Results:**

From 1992 to 2015, the annual number of visits per practitioner decreased by 23%. The number of patients per practitioner who received fillings, crowns, bridges, dentures, root fillings, or extractions decreased by 50% or more. The decrease was largest for practitioners younger than 35 years and for men. The proportion of practitioners who reported a deficit of patients increased from 20% to 37%.

**Conclusions:**

Many dentists will have too few patients and a fall in income in the years to come is expected.

## Introduction

There has been a marked improvement in dental health in most Western countries during the last few decades.^1^, ^2^, ^3^, ^4^, ^5^, ^6^, ^7^ Several reasons have been suggested, the most important of which are fluoride toothpaste and better oral hygiene.^3^^,^^5^^,^^8^ Even though policy makers agree that dental health has improved, policy changes have not been made to take account of the implications.

The dominant view during the end of the last century was that improvements in dental health would lead to less demand for dental care.^2^^,^^9^, ^10^, ^11^, ^12^ If the supply of dentists was not adjusted accordingly, this could have serious consequences for the dental profession. In several countries, the supply of dental manpower has not been adjusted to the lower demand.^13^, ^14^, ^15^, ^16^ For example, in the Scandinavian countries the number of inhabitants per dentist has been high, slightly above 1000 for the last few decades.^13^^,^^15^, ^16^ With less demand for care and no adjustment of supply of dental manpower, dentists may experience a deficit of patients. Thus, fewer dentists should be trained.

In light of the improvements in dental health in Norway, the focus of the present work was to examine the following: How has provision of dental services changed, and how has private dental practitioners’ assessment of their workload changed? The data were from 2 large surveys of private dental practitioners carried out in 1992 and 2015. During this time, there was a marked improvement in dental health in the Norwegian population and the number of inhabitants per dentist was about 1100.^17^, ^18^, ^19^, ^20^, ^21^, ^22^ If there was less demand for dental care in 2015 than in 1992 due to improvements in dental health, then it would be expected that the proportion of practitioners who reported a deficit of patients would increase.

First, the organisation of private dental practice in Norway is described. In the sections that follow, the data and the outline for the analyses is presented. In the last 2 sections, the results are presented and some policy implications of the findings are discussed.

## Dental services in Norway

Dental services in Norway are divided into a public and a private sector. The public dental service has responsibility for providing dental care for all children and young people up to the age of 18, mentally handicapped people , and elderly people who receive care in an institution or home nursing care.^23^ About 30% of dentists work in the public dental service.^17^ Public dental services are free to patients. Public dental officers are employed by the county municipalities and receive a fixed salary.

Adults receive dental treatment from private dental practitioners. There are approximately 4 million people in Norway aged 20 years and older, of whom nearly 80% say that they have been to the dentist for a regular checkup during the last year.^24^^,^^25^ From November 15, 1995, a deregulated fee system for supply of dental services for people aged 20 years and older was introduced. Up until this time, dental fees were determined through annual negotiations between the Ministry of Government Administration and the Norwegian Dental Association. After the deregulation, private dental practitioners could freely advertise their services. Private dental services for adults are almost entirely financed by patient fees. Reimbursements from the National Insurance Administration are limited. Private dental practitioners have the freedom to establish a practice where they want to. Only the number of dental students is regulated by the government. There are few health services in Western Europe that are so strongly deregulated as private dental services in Norway.^26^

## Material and methods

### Sample

The data for this study were collected using a questionnaire that was sent to all private dental practitioners at the end of 1992 and 2015. The surveys were a joint project with the Norwegian Dental Association and the Department of Community Dentistry, University of Oslo. In 1992, the survey was carried out by the poll company Scan-Fact A/S and in 2015 by the Norwegian Dental Association. All responses were treated anonymously.

Nearly all private dental practitioners are members of the Norwegian Dental Association. The questionnaires were sent to the members who worked in private dental practice, with the exception of specialists and dentists who were not currently working, for example, dentists on maternity leave or study leave.

In 1992, the questionnaire was sent to 1916 private dental practitioners. Altogether, 1056 responded, which gave a response rate of 55%. In 2015, the questionnaire was sent to 2214 private dental practitioners. Altogether, 1237 responded, which gave a response rate of 56%. An analysis of nonresponders showed that they were evenly distributed according to age, gender, and the region in which their practice was located (Appendix A). Thus, the samples were representative of private dental practitioners. In 1992, 48 private dental practitioners had filled out the questionnaire incorrectly or incompletely. In 2015, this figure was 91. These dentists were excluded from the study population.

### Variables

#### Provision of services

For 1 representative week in practice, the private dental practitioners were asked to report the number of visits and the number of patients who received one or more of the following items of treatment: filling, crown, bridge, denture, root filling, extraction, and periodontal treatment. The private dental practitioners chose a representative week on the grounds that they had no activities that kept them out of the dental practice (eg, holiday, courses, meetings). Estimates for the year were calculated by multiplying the figures for 1 week by the number of weeks worked during the year.

#### Assessment of workload and characteristics of the practitioner and the practice

As a measure of private dental practitioners’ supply of patients, the responses from the following questions were used: “Based on an overall assessment of economy, workload, and other personal factors, is the number of regular patients adequate? If not, do you wish to have more patients or fewer patients?” For those who wished to have more patients or fewer patients, they were also asked to report how many more or fewer. Information was collected about the private dental practitioners’ age and gender.

### Analyses

The following were analyzed:

a) Changes in provision of dental services from 1992 to 2015. This was done by comparing the mean values in 1992 and 2015 for number of visits and types of treatment.
b) Changes in the proportion of private dental practitioners who reported a deficit of patients from 1992 to 2015.

The comparisons in a) and b) were carried out for the whole sample and in subsamples according to the private dental practitioners’ age and gender.

### Supplementary analyses using survey data from patients

Data on diagnostic and preventive services are not available from surveys of private dental practitioners. However, such data are available from population surveys that are representative of the adult Norwegian population. These surveys have shown that 77% of people aged 20 years and older have regular checkups annually and 11% have regular checkups every 2 years.^24^^,^^25^ A checkup includes an examination for caries and periodontal disease. The respondents were also asked whether they received other preventive services: removal of plaque and calculus, application of fluorides, oral hygiene instruction, and dietary advice.

In our supplementary analyses, survey data was used to analyse changes in provision of preventive services from 1991 to 2013. The comparisons between 1991 and 2013 were carried out for the whole sample and in subsamples according to age and gender. In 1991, the sample included 3359 persons, and in 2013, 5422 persons were included. For details about the sampling procedure and the representativeness of the samples, see Appendix B and Grytten et al.^25^

## Results

### Changes in provision of dental services from 1992 to 2015

During the period from 1992 to 2015, the annual number of visits per private dental practitioner decreased from 2634 to 2030, that is, by 23% (Table 1). The decrease was largest for practitioners younger than 35 years and for men. For each age group and for gender, none of the 95% confidence intervals overlapped in 1992 and 2015.

**Table 1:** Number of visits per private dental practitioner according to age and gender.

Characteristics of the private dental practitioner	Number of visits		Percentage reduction in number of visits
	1992 (n = 1008)	2015 (n = 1146)	
**Age**			
<35 years	2382	1722	-28
	[2236-2529]	[1621-1823]	
35-49 years	2718	2049	-25
	[2618-2819]	[1983-2116]	
≥50 years	2714	2144	-21
	[2614-2813]	[2072-2216]	
**Gender**			
Men	2749	2183	-21
	[2678-2820]	[2120-2245]	
Women	2137	1835	-14
	[2006-2268]	[1774-1896]	
			
**Mean**	2634	2030	-23
	[2570-2699]	[1985-2075]	

Annual figures. 95% confidence intervals in brackets. 1992 and 2015.

In 1992, practitioners who were younger than 35 years had 2382 visits annually. In 2015, these practitioners would be around 50 years or older. In 2015, the number of visits for practitioners in the age group 50 years or older was 2144. For these 2 age groups, none of the 95% confidence intervals overlapped in 1992 and 2015. Thus, during a working life of more than 20 years in practice, patient supply decreased.

There was a marked decrease in the number of patients per practitioner who received fillings, crowns, bridges, dentures, root fillings, or extractions from 1992 to 2015 (Table 2). For most of these items of treatment, the decrease was around 50% or more. None of the 95% confidence intervals overlapped in 1992 and 2015. For periodontal treatment, the number of patients increased from 1992 to 2015.

**Table 2:** Mean number of patients who received different types of treatment per private dental practitioner.

Types of treatment	1992 (n = 1008)		2015 (n = 1146)		Percentage annual change from 1992 to 2015
	Mean number of patients per year	Mean number of patients per week	Mean number of patients per year	Mean number of patients per week	
Fillings	1342	29.9	719	16.5	-46
	[1293-1390]	[28.8-30.9]	[692-745]	[16.0-17.1]	
Crowns	140	3.1	97	2.2	-31
	[132-147]	[2.9-3.3]	[91-102]	[2.1-2.3]	
Bridges	48	1.1	22	0.5	-54
	[44-52]	[1.0-1.2]	[20-23]	[0.47-0.54]	
Denture, including repair	51	1.1	14	0.3	-72
	[47-54]	[1.0-1.2]	[13-15]	[0.29-0.35]	
Implants			2.8	0.06	
			[2.1-3.5]	[0.05-0.08]	
Root filling	169	3.8	77	1.8	-54
	[160-178]	[3.6-4.0]	[73-82]	[1.7-1.9]	
Extraction	132	2.9	59	1.4	-55
	[122-142]	[2.7-3.1]	[56-63]	[1.3-1.5]	
Periodontal treatment	46	1.0	83	1.9	81
	[33-59]	[0.7-1.3]	[76-90]	[1.8-2.1]	

Figures per year and per week. 95% confidence intervals in brackets. 1992 and 2015.

In 2015, the number of patients per week who needed a crown, bridge, denture, root filling, or extraction was in the range 0.3 to 2.2. Most patients needed fillings. On average, the practitioners treated 16.5 such patients per week (Table 2).

### Deficit of patients from 1992 to 2015

During the period from 1992 to 2015, the proportion of practitioners who reported a deficit of patients increased from 20% to 37% (Table 3). The proportion of practitioners who reported a surplus of patients decreased from 10% to 3% during the same period. For both years, the dentists who reported a deficit of patients had the fewest visits and those who reported a surplus had the most visits.

**Table 3:** Proportion of private dental practitioners and mean number of visits per year according to assessment of workload.

	1992 (n = 1008)		2015 (n = 1146)	
Assessment of workload	Percentage private dental practitioners	Mean number of visits per year	Percentage private dental practitioners	Mean number of visits per year
Satisfied with the number of patients	70	2713	60	2172
	[67-73]	[2639-2787]	[57-63]	[2116-2229]
Deficit of patients	20	2117	37	1777
	[18-23]	[1989-2247]	[34-40]	[1706-1847]
Surplus of patients	10	3127	3	2325
	[18-12]	[2894-3360]	[2-4]	[2036-2614]

95% confidence intervals in brackets. 1992 and 2015.

The increase in the proportion who reported a deficit was largest for practitioners younger than 35 years and for women (Table 4). For each age group and for gender, none of the 95% confidence intervals overlapped in 1992 and 2015.

**Table 4:** Proportion of private dental practitioners who reported a deficit of patients according to age and gender.

Characteristics of the private dental practitioner	1992 (n = 1008)	2015 (n = 1146)	Number of additional patients the private dental practitioner wished in 2015
**Age**			
<35 years	33	54	439
	[26-40]	[47-60]	[384-494]
35-49 years	18	38	404
	[14-21]	[34-43]	[356-452]
≥50 years	16	29	303
	[12-19]	[25-33]	(262-344]
**Gender**			
Men	19	34	400
	[17-22]	[31-38]	[354-445]
Women	23	41	360
	[17-29]	[37-45]	[327-392]
			
**Mean**	20	37	380
	[18-23]	[34-40]	[352-408]

Percentages. 95% confidence intervals in brackets. 1992 and 2015.

In 1992, 33% of practitioners who were younger than 35 years reported a deficit of patients (Table 4). In 2015, these practitioners would be around 50 years or older. In 2015, 29% of the practitioners in the age group 50 years and older reported a deficit of patients. For these 2 age groups, the 95% confidence intervals overlapped in 1992 and 2015. Thus, during a working life of more than 20 years in practice, practitioners’ assessment of workload was the same.

In 2015, the proportion of practitioners who reported a deficit of patients was particularly high in the youngest age group and for women. The mean number of additional patients that practitioners younger than 35 years wished to have was 439. The corresponding figure in the age group 50 and older was 303 (Table 4). The 95% confidence intervals did not overlap.

### Supplementary analyses

In Appendix C, we present the results from the analyses of the population surveys. Only participants who had visited the dentist during the last year were included. During the period from 1991 to 2013, the proportion of patients who received preventive services increased from 57% to 88%. The increase was in the same order of magnitude for all age groups and for men and women. None of the 95% confidence intervals overlapped in 1991 and 2013.

## Discussion

**Fig. 1:**
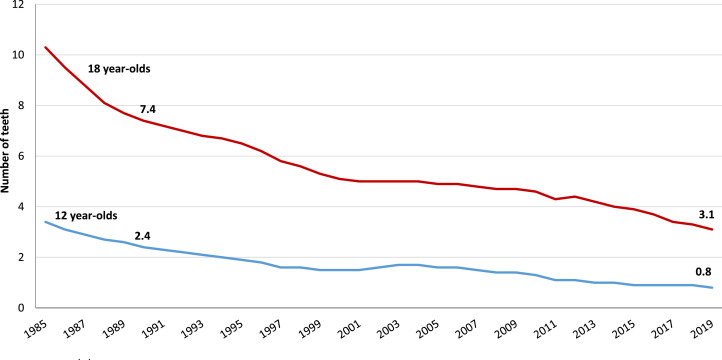
Improvement in dental health amongst children and adolescents in Norway. Mean number of teeth with caries experience (DMFT). 1985-2019.

There has been a marked improvement in dental health in Norway during the last few decades.^17^, ^18^, ^19^, ^20^, ^21^^,^^27^ For example, the mean number of decayed, missing and filled teeth (DMFT) for 18 year-olds decreased from 7.4 in 1990 to 3.1 in 2019 (Figure 1)^17^. Nearly all 18-year-olds had 1 or more teeth with fillings or caries in 1990. Thirty years later, 30% of them were caries-free. This reduced demand has continued into adult life.^18^^,^^20^^,^^21^ This is why such a large proportion of practitioners with a deficit of patients was found. Treatment need amongst elderly people has also declined during the last few decades.^18^^,^^19^^,^^21^ This has also contributed to the increase in the proportion of practitioners with a deficit of patients.

There are few studies that have examined the implications that improvements in dental health have had on provision of dental services. Such studies are important, as they provide insight into whether dentists adjust their provision of services according to changes in the dental health of the population. Our results show that such adjustments do take place. Due to improvements in dental health, from 1992 to 2015 there was a marked decrease in the mean number of visits and the mean number of patients who received different types of treatment per practitioner (Tables 1 and 2). The decrease was largest for practitioners younger than 35 years. Patient supply also decreased over time for dentists who were in the youngest age group in 1992. In other words, these dentists did not manage to increase their patient base as they became older.

Most of the existing studies within this field are from Australia.^28^, ^29^, ^30^, ^31^ In these studies, the data were collected from private dental practitioners every fifth year from 1983 to 2009. The sample sizes were in the range of 309 (in 1983) to 561 (in 2009).^28^ The results presented here are in line with most of the findings from the Australian studies. For example, in the Australian studies there was a decline in the annual number of visits per practitioner over time.^29^ The proportion of patients who received restorative, oral surgical, and prosthodontic treatment also decreased over time.^30^^,^^31^ Diagnostic and preventive services or crowns increased from 1982 to 2010.^30^^,^^31^ The data presented here did not have information about diagnostic and preventive services. However, patient surveys have shown that diagnostic services have also increased in Norway from 1973 to 2013.^25^^,^^32^ This was also the case for preventive services, as shown in the supplementary analyses (Appendix C).

It is interesting that the trends in provision of dental services are similar in Norway and Australia, even though several characteristics of the dental care market are different in these countries. In Australia, the oral health system is almost entirely a private system. The social gradient in Australia is steeper than in Norway, and a large proportion of the population cannot afford dental care.^33^, ^34^, ^35^, ^36^ The other difference is that the dentist-to-population ratio in Australia has increased relatively drastically over the years due to more dental schools being established.^35^

In 2015, there were certain types of treatment that practitioners seldom provided, such as bridges, dentures, and implants (Table 2). For patients, it is important that dentists are highly skilled in the treatment procedures they carry out. Are dentists more highly skilled in procedures that they carry out often? Is the quality of treatment provided for these procedures higher? There is evidence that this is the case for medical procedures. Several studies have shown a lower mortality rate in hospitals where many procedures are performed (high-volume hospitals) compared to hospitals where few procedures are performed (low-volume hospitals).^37^, ^38^, ^39^ Taking this evidence into account, it is suggested that patients who need types of treatment that private dental practitioners seldom provide should be referred to dental specialists. In Norway, there are several specialities, such as oral surgery, prosthetics, periodontics, and endodontics.^40^ Within each speciality, the number of specialists per capita is high, and studies have shown that specialist services are easily available.^17^^,^^41^ An argument against referring patients to specialists is that the treatment would be more expensive and thus may lead to inequalities in access. In the long run, it would also lead to fewer patients for dentists. Thus, it may be even more difficult for dentists to maintain their skills in procedures that they seldom carry out.

The results from this study have implications for future manpower policies for oral health care in Norway. Such policies have been developed by Statistics Norway on the basis of workforce planning models.^42^ These models involve comparing the expected future supply of dentists with the expected future demand for dental care. Future demand has been forecast on the basis of demographic changes, such as the size and age distribution of the population. According to the most recent estimation, the number of human-labour years for dentists is expected to increase from 4600 to 5800 by the year 2035.^42^ This increase will occur because the number of newly qualified dentists is larger than the number of dentists leaving the profession (mostly due to retirement). Towards 2035, the rate of population growth will be less than the rate of growth for the number of dentists. Thus, without any changes in manpower policies, in 2035 the population:dentist ratio will be about 1000 or even less.

In the next few decades, the proportion of elderly people in the population will increase. For example, the proportion of people aged 70 years and older will increase from 12% in 2020 to 17% in 2035.^43^ It can be expected that treatment needs will be greater for elderly people than for younger people. Elderly people are maintaining their dentitions for longer, and age-associated oral health conditions will pose new challenges, especially combined with polypharmacy in older adults. Today, nearly 40% of dentists have a deficit of patients. The population:dentist ratio is about 1100. Thus, with a population:dentist ratio of about 1000 in 2035, there would be more than enough dentists to meet increased demand amongst elderly people. In fact,it can be expected that the number of practitioners with a deficit of patients will continue to increase from today's level. Several factors point in that direction.

First, it is likely that dental health will continue to improve, and that this will lead to a further reduction in demand for dental care. This is not to say that the entire population will be caries-free, but taking present trends into account, treatment needs will be less in the future. We can expect that there will always be a need for some types of dental treatment, for example, orthodontic treatment.

Second, during the next few decades, the pre-fluoride generation will have died out. People in this generation, born before 1970, have large restorations and many missing teeth.^44^, ^45^, ^46^, ^47^ Maintaining the dentitions of these people has been a major source of revenue for practitioners during the last few decades. Those born after 1970, the post-fluoride generation, have fewer restorations that may need to be repaired later in life.^44^, ^45^, ^46^, ^47^ Meeting the treatment needs of the post-fluoride generation will contribute less to practitioners’ income compared to what they earned from treating the pre-fluoride generation.

Third, the recall interval may become longer in the future. Patients who have little dental disease may not feel that short recall intervals are necessary. For example, in Norway the most frequent recall interval in the age group 20 to 39 years is currently 24 months, compared to 12 months in 2004.^24^^,^^25^

Fourth, provision of more preventive services is unlikely to result in an increase in workload. Most adults already receive such services (Appendix C). Also, if preventive services are effective, this would result in a further decrease in workload.

Fifth, the number of human-labour years for dental hygienists is expected to increase from 1100 to 1600 by the year 2035.^42^ Dental hygienists have the legal right to provide diagnostic and preventive services.^48^ From the beginning of this century, there has been a large increase in the number of dental hygienists who work in the public and private dental care sectors (Appendix D). Dental practitioners diagnose oral diseases, but dental hygienists have had increasing responsibility for diagnosing oral diseases. This will result in a further decrease in workload for dentists.

Sixth, during the last few decades, there has been a marked increase in the level of education in Norway.^49^ The proportion of persons aged 30 to 39 years with university/college education increased from 20% in 1980 to 50% in 2019 (see Appendix E and Statistics Norway^49^). There is a strong positive association between education and dental health behaviour and between education and dental health (for a review, see Grytten^50^). Norwegians have favourable dental health behaviour in relation to oral hygiene and sugar consumption (see Appendix F and Søgaard et al.^51^). The level of education in the population is expected to continue to increase, and Norwegians are likely to maintain their favourable dental health behaviour in the years to come. This will lead to a further improvement in dental health and thereby a further decrease in workload for dentists.

There is already a substantial surplus of dentists, and most likely this will continue to increase. This is not a problem as long as normal market mechanisms operate on the dental care market. However, to examine whether the dental care market operates as a perfect competitive market was not the aim of the present study. Within the field of health economics, there is a large amount of literature in which this research question has been examined (for a review, see Greenberg,^52^ Frech,^53^ Carlsen and Grytten,^54^ and McGuire^55^). The underlying idea for this research is that if physicians' income is threatened, then they can counteract a fall in income by increasing the quantity and/or the fee for the services they offer. Physicians' income may fall due to a decline in demand for their services and/or due to an increase in supply of physicians per capita. Rather than reducing their fees to attract more patients, physicians may raise their fees and/or increase the quantity of services they provide to make up for lost revenue.

Within dentistry, there are a few studies that have examined whether dentists have market power. These studies show that dentists are able to counteract a fall in demand for their services by raising their fees, by recalling their patients more often, and by increasing the cost per visit.^26^^,^^56^, ^57^, ^58^, ^59^, ^60^, ^61^ This is because the dental care market does not operate as a perfect competitive market, that is, there is an absence of a demand function that is independent of supply-side influences.^26^^,^^62^

From a policy point of view, the key question is what to do about the surplus of dentists. There are two options. The first option is to let the marketplace solve the problem. Less demand and more market competition will lead to declining income for practitioners. Most likely, this will be the case even though practitioners may compensate for some of the income loss by raising their fees, by recalling their patients more often, and by increasing the cost per visit. Decline in income is expected to result in fewer applicants to dental schools, and with fewer applicants, some dental schools may close. The market should be able to clear the excess capacity of dentists, but this would take several years. The second option is for central authorities to reduce enrolment of students to dental schools straightaway. Training dentists takes about five years. If the number of dentists trained were reduced now, this would not make a significant difference to the total stock of dentists until a few years ahead. However, this process would probably go faster than using market competition. Whatever policy is introduced, many dentists will have too few patients and a subsequent fall in income in the years to come.

## Conclusions

A large decrease in the number of visits and different types of treatment from 1992 to 2015 was found. The reason for this decrease is the marked improvement in dental health. Dental health will probably continue to improve, and this will lead to a further reduction in treatment needs.

The proportion of practitioners who reported a deficit of patients increased markedly from 1992 to 2015. In 2015, nearly 40% of all private dental practitioners had a deficit of patients. Most likely, many dentists will have too few patients and a fall in income in the years to come is expected.

## Conflict of interest

None disclosed.

## Appendix Supplementary materials

Image, application 1

## Footnotes

Supplementary material associated with this article can be found in the online version at doi:10.1016/j.identj.2021.07.004.
