# Supplementary material for: Improvements in Dental Health and Dentists’ Workload in Norway, 1992 to 2015
Source: Int Dent J. 2021 Sep 1;72(3):399–406. doi: 10.1016/j.identj.2021.07.004 (PMC9275092; doi:10.1016/j.identj.2021.07.004)
Supplement: Supplementary file 1 [file mmc1.docx]

**APPENDICES**

**Appendix A**

Private dental practitioners. Description of the sample and representativeness in relation to the population. Per cent

|  |  |  |  |  |  |  |
| --- | --- | --- | --- | --- | --- | --- |
|  |  | 1992 | |  | 2015 | |
|  |  | Gross sample^1^ | Net sample^2^ |  | Gross sample^1^ | Net sample^2^ |
|  |  | (n=1 916) | (n=1 056) |  | (n=2 214) | (n=1 237) |
|  |  |  |  |  |  |  |
|  |  |  |  |  |  |  |
| **Gender** | |  |  |  |  |  |
|  | Women | 23 | 19 |  | 47 | 46 |
|  | Men | 77 | 81 |  | 53 | 54 |
|  |  |  |  |  |  |  |
| **Age** | |  |  |  |  |  |
|  | < 35 years | 24 | 21 |  | 21 | 19 |
|  | 35 - 49 years | 41 | 42 |  | 40 | 40 |
|  | >= 50 years | 35 | 37 |  | 39 | 41 |
|  |  |  |  |  |  |  |
| **Region** | |  |  |  |  |  |
|  | Eastern Norway, ex. Oslo region | 28 | 29 |  | 26 | 27 |
|  | Oslo region | 31 | 28 |  | 30 | 28 |
|  | Southern and Western Norway | 29 | 29 |  | 30 | 32 |
|  | Mid- and Northern Norway | 12 | 14 |  | 14 | 13 |
|  |  |  |  |  |  |  |
|  |  |  |  |  |  |  |
| ^1^ Number of private dental practitioners who were asked to participate | | | |  |  |  |
| ^2^ Number of private dental practitioners who actually participated | | | |  |  |  |

**Appendix B**

SURVEY DATA 1991 and 2013.

*Sampling procedure*

The data were collected using a postal questionnaire that was sent by TNS Gallup in 1991 and 2013 to Norwegians aged 20 years or older living at home. For each year, the sample was obtained in the following way: The adult Norwegian population was first stratified according to age, gender and region. Within each stratum, a proportional and random sample of persons was drawn in order to select enough respondents. This is not the type of sample for which it is appropriate to give a drop-out rate. When people in the population do not want to be in the sample, TNS Gallup select more persons at random until the sample contains the required number of people (n= 3 359 in 1991, and n=5422 in 2013). Much work is carried out to make the sample as representative of the adult population as possible. So the representativeness of the sample is good.

The distribution of the samples according to gender, age and region was about the same as the distribution in the population (see table below). In order to improve the representativeness of the sample even further, it was weighted. The distribution of the population according to gender, age and region was used to construct the sampling weights.

Reference:

1. Fladmoe A. Tannhelsetjenester i Norge. Fakta om undersøkelsen. TNS-Gallup, Mai 2013.

*Description of the samples and representativeness in relation to the population.*

*Survey data 1991 and 2013. Per cent*

|  |  |  |  |  |  |  |
| --- | --- | --- | --- | --- | --- | --- |
|  |  | 1991 | |  | 2013 | |
|  |  | Sample  (n=3 359) | Population 20 years and older ^1^ |  | Sample  (n=5 422) | Population 20 years and older ^1^ |
|  |  |  |  |  |  |  |
| Gender | |  |  |  |  |  |
|  | Women | 50.2 | 50.5 |  | 54.3 | 49.8 |
|  | Men | 49.8 | 49.5 |  | 45.7 | 50.2 |
|  |  |  |  |  |  |  |
| Age (in years) | |  |  |  |  |  |
|  | 20-34 | 38.4 | 31.4 |  | 22.6 | 26.5 |
|  | 35-44 | 20.6 | 20.0 |  | 16.7 | 19.0 |
|  | 45-54 | 14.3 | 14.0 |  | 15.9 | 18.1 |
|  | 55-64 | 11.3 | 12.4 |  | 18.4 | 15.6 |
|  | >=65 | 15.3 | 22.2 |  | 26.3 | 20.8 |
|  |  |  |  |  |  |  |
| Region | |  |  |  |  |  |
|  | Eastern Norway | 51.0 | 48.8 |  | 49.7 | 50.1 |
|  | Southern Norway | 5.2 | 5.7 |  | 5.4 | 5.7 |
|  | Western Norway | 24.4 | 25.7 |  | 25.3 | 26.1 |
|  | Trøndelag | 8.8 | 8.9 |  | 9.8 | 8.7 |
|  | Northern Norway | 10.6 | 10.9 |  | 9.6 | 9.4 |
|  |  |  |  |  |  |  |
|  |  |  |  |  |  |  |
| ^1^ Source: Statistics Norway | | |  |  |  |  |

**Appendix C**

Proportion of patients who received preventive services. Patients who had visited the dentist during the last year. Per cent. 95% confidence intervals in brackets. Surveys 1991 and 2013

|  |  |  |  |  |
| --- | --- | --- | --- | --- |
| Characteristics of patients | | 1991  (n=2 493) Per cent |  | 2013 (n=4 333) Per cent |
|  |  |  |  |  |
| **Age** | |  |  |  |
|  | 20-34 years | 52 |  | 82 |
|  |  | [49 - 56] |  | [79 - 85] |
|  | 35-44 years | 61 |  | 91 |
|  |  | [57 - 65] |  | [89 - 93] |
|  | 45-54 years | 62 |  | 91 |
|  |  | [57 - 66] |  | [89 - 93] |
|  | 55-64 years | 63 |  | 91 |
|  |  | [58 - 69] |  | [90 - 93] |
|  | >=65 years | 57 |  | 87 |
|  |  | [51 - 63] |  | [85 - 89] |
|  |  |  |  |  |
| **Gender** | |  |  |  |
|  | Men | 60 |  | 89 |
|  |  | [57 - 63] |  | [88 - 91] |
|  | Women | 56 |  | 88 |
|  |  | [53 - 58] |  | [86 - 89] |
|  |  |  |  |  |
|  |  |  |  |  |
| Mean | | 57 |  | 88 |
|  |  | [55 - 59] |  | [87 - 89] |
|  |  |  |  |  |
|  |  |  |  |  |
|  |  |  |  |  |

**Appendix D**

Man-labour years for dental hygienists according to year.

|  | Type of dental care sector | |
| --- | --- | --- |
| Year | Public | Private |
|  |  |  |
| 2002 | 316.2 | 223.0 |
| 2003 | 352.0 | 224.6 |
| 2004 | 349.1 | 259.9 |
| 2005 | 366.1 | 272.5 |
| 2006 | 382.0 | 296.0 |
| 2007 | 388.8 | 325.1 |
| 2008 | 402.2 | 350.6 |
| 2009 | 410.4 | 324.5 |
| 2010 | 431.0 | 342.8 |
| 2011 | 438.8 | 386.7 |
| 2012 | 454.1 | 379.6 |
| 2013 | 468.9 | 390.5 |
| 2014 | 459.7 | 403.6 |
| 2015 | 511.1 | 407.8 |
| 2016 | 519.3 | 429.4 |
| 2017 | 532.1 | 457.4 |
| 2018 | 537.8 | 460.4 |
| 2019 | 552.7 | 480.4 |
| 2020 | 559.7 | 483.8 |
|  |  |  |
|  |  |  |
| Per cent increase 2002-2020 | 77 | 117 |
|  |  |  |
|  |  |  |
| Source: Statistics Norway. Statbank. Dental health care | | |

**Appendix E**

Proportion of persons aged 30-39 years with university/college education according to year.

Norway 1980 – 2019

Source: Statistics Norway

**Appendix F**

Sugar consumption per person per year (kg). Norway 2000-2020
